# Retrospective analysis on efficacy of convalescent plasma in acute respiratory distress syndrome due to COVID-19

**DOI:** 10.1590/1516-3180.2021.0200.R1.03052021

**Published:** 2021-08-27

**Authors:** Esma Eren, Ayşegül Ulu-Kılıç, Serdal Korkmaz, Merve Yıldız, Recep Civan Yüksel, Ayşin Kılınç-Toker, Emine Arman-Fırat, Zehra Bestepe-Dursun, Ilhami Çelik

**Affiliations:** I MD. Physician, Department of Infectious Diseases and Clinical Microbiology, University of Health Sciences, Kayseri City Education and Research Hospital, Kayseri, Turkey.; II MD, PhD. Professor, Department of Infectious Disease and Clinical Microbiology, Erciyes Üniversitesi Tıp Fakültesi, Kayseri, Turkey.; III MD, PhD. Assistant Professor, Department of Hematology, University of Health Sciences, Kayseri City Education and Research Hospital, Kayseri, Turkey.; IV MD. Physician, Department of Infectious Diseases and Clinical Microbiology, University of Health Sciences, Kayseri City Education and Research Hospital, Kayseri, Turkey.; V MD. Physician, Intensive Care Unit, University of Health Sciences, Kayseri City Education and Research Hospital, Kayseri, Turkey.; VI MD. Physician, Department of Infectious Diseases and Clinical Microbiology, University of Health Sciences, Kayseri City Education and Research Hospital, Kayseri, Turkey.; VII MD. Physician, Department of Infectious Diseases and Clinical Microbiology, University of Health Sciences, Kayseri City Education and Research Hospital, Kayseri, Turkey.; VIII MD. Physician, Department of Infectious Disease and Clinical Microbiology, Kayseri City Education and Research Hospital, Kayseri, Turkey.; IX MD, PhD. Professor, Department of Infectious Diseases and Clinical Microbiology, University of Health Sciences, Kayseri City Education and Research Hospital, Kayseri, Turkey.

**Keywords:** COVID-19, Respiratory distress syndrome, COVID-19 serotherapy [supplementary concept], Coronavirus disease 2019, Convalescent plasma, Acute respiratory distress syndrome.

## Abstract

**BACKGROUND:**

Coronavirus disease 2019 (COVID-19) is an ongoing global health threat. However, currently, no standard therapy has been approved for the disease.

**OBJECTIVES:**

To evaluate the clinical effectiveness of convalescent plasma (CP) in patients with acute respiratory distress syndrome (ARDS) due to COVID-19.

**DESIGN AND SETTING:**

Retrospective study conducted at Kayseri City Education and Research Hospital, Kayseri, Turkey.

**METHODS:**

The case group consisted of adult patients (> 18 years) with ARDS due to COVID-19 who received CP in combination with antiviral and supportive treatment. These patients were compared with others who only received antiviral and supportive treatment.

**RESULTS:**

During the study period, a total of 30 patients with ARDS due to COVID-19 were included. Eleven patients (36%) received CP in combination with antiviral and supportive treatment, whereas nineteen patients (64%) in the control group only received antiviral and supportive treatment. On admission, the median age, demographic and clinical data and initial laboratory test results were similar between the groups (P > 0.05). On the 14^th^ day of treatment, the laboratory values remained similar between the groups (P > 0.05). The mortality rates were not significantly different between the groups.

**CONCLUSION:**

CP treatment did not affect mortality or lead to clinical improvement for COVID-19 patients with ARDS.

## INTRODUCTION

At the end of 2019, a novel coronavirus was recognized as a cause of viral pneumonia cases in Wuhan, China. Since it had high genetic similarity to severe acute respiratory syndrome coronavirus (SARS-CoV), the virus was officially named SARS-CoV-2 and the disease was named coronavirus disease 2019 (COVID-19).^1^ The disease spread in a short time and was declared to be a pandemic by the World Health Organization (WHO) on March 11, 2020.^2^ Globally, there have been 119,603,761 confirmed cases of COVID-19, including 2,649,722 deaths, reported to WHO, starting from the day on which it was first identified.^3^

Currently, there is no proven effective treatment for COVID-19.^4^ Convalescent plasma (CP) treatment is one of the passive immunotherapy methods used. This is a very old procedure.^5^ It was used successfully against SARS-CoV in 2003, the influenza A pandemic (H1N1) in 2009, Middle East respiratory syndrome coronavirus in 2012, and Ebola virus in 2015. Mair-Jenkins reviewed the experiences from all of these epidemics and reported that CP therapy was associated with reduced mortality.^6^

The use of CP for treating COVID-19 is highly controversial.^7^ Ye et al. administered CP to six patients infected with SARS-CoV-2 and reported a rapid and dramatic improvement in patients who presented lung infiltration.^8^ It was also reported that CP improved two patients with acute respiratory distress syndrome (ARDS) due to COVID-19.^9^

In a systematic review, CP was shown to have reduced the mortality rate among high-risk COVID-19 patients.^10^ On the other hand, in another study, a meta-analysis comparing CP therapy with placebo did not demonstrate any evidence of benefit from use of CP compared with placebo or standard care, with regard to clinical improvement or reduction of all-cause mortality.^11^

## OBJECTIVE

In this present study, we aimed to evaluate the effectiveness of CP for patients with ARDS due to COVID-19.

## METHODS

### Study design and patients

This retrospective study was carried out in a tertiary-level hospital with a total capacity of 1,600 beds, and 180 intensive care beds. Adult patients (> 18 years) who were treated in the intensive care unit (ICU) for ARDS due to COVID-19 were included to this study. The case group was defined as patients who received CP in addition to antiviral and supportive treatment. The control group only received antiviral and supportive treatment. The primary endpoint of the study was 14-day mortality; the secondary endpoints were improvement in respiratory function (PaO_2_/FiO_2_ ratio) and the laboratory findings on the 14^th^ day of the disease.

### Definitions

Presence of COVID-19 pneumonia was defined as either: I. SARS-CoV-2 PCR positivity in upper respiratory tract samples and bilateral peripheral ground-glass infiltration (typical for COVID-19) in thoracic computed tomography (CT) scans; or: II. Rapid antibody test positivity and typical infiltration for COVID-19 in thoracic CT.

ARDS was defined in accordance with the Berlin criteria: I. Respiratory distress that occurred or worsened in the last seven days; II. Radiologically detected pleural effusion, lung collapse or bilateral nodular opacities; III. Respiratory failure that cannot be explained by heart failure or volume; and IV. Hypoxemia. ARDS were classified according to the degree of hypoxemia, as mild ARDS (200 < PaO_2_/FiO_2_ ≤ 300; positive end-expiratory pressure, PEEP ≥ 5 cmH_2_O); moderate ARDS (100 < PaO_2_/FiO_2_ ≤ 200; PEEP ≥ 5 cmH_2_O); or severe ARDS (PaO_2_/FiO_2_ ≤ 100; PEEP ≥ 5 cmH_2_O), according to oxygenation.^12^

Patients who had a diagnosis of cancer, were receiving any immunosuppressive therapy or had serum IgA deficiency, and pregnant women, were excluded from the study.

### Institutional protocol for treating COVID-19 patients

For COVID-19 patients who did not need hospitalization or had mild symptoms, oral hydroxychloroquine and oral azithromycin were prioritized. All critically ill patients were treated with favipiravir. ABO-compatible plasma was used for patients from eligible donors or if the blood type-compatible plasma was already available in the blood center of the hospital. Supportive therapy consisted of oxygen and fluid supplements, and also vasopressor agents if necessary. All patients received favipiravir (1600 mg loading dose and 800 mg/day maintenance dose, orally), methylprednisolone (40-80 mg/day parenterally) and enoxaparin (4,000-6,000 IU).

### Selection of CP donors

The criteria for selecting individuals as donors were as follows: I. Evidence of COVID-19 documented by a laboratory test, consisting either of a diagnostic test (e.g. on a nasopharyngeal swab) at the time of illness, or of a positive serological test for SARS-CoV-2 antibodies after recovery, if prior diagnostic testing was not performed at the time when COVID-19 was suspected; II. Complete resolution of symptoms at least 14 days prior to donation and negative results for COVID-19 either from one or more nasopharyngeal swab specimens or from a molecular diagnostic test on a blood sample.^13^

Individuals who had recovered from SARS-CoV-2 infection were invited to donate CP. All donors were informed about the apheresis procedure, and their written consent was obtained. In addition to a RT-PCR assay for SARS-CoV-2 RNA, all potential donors were serologically screened for HBsAg, anti-HCV, anti-HIV 1-2 and anti-syphilis antibodies. The neutralizing antibody titer was not routinely obtained. The latest generation of cell separator apheresis device (Spectra Optia apheresis system; Terumo BCT, Lakewood, United States) was used to collect CP. 200-600 milliliters (ml) of plasma were collected using the apheresis device, depending on the total blood volume of the donor. Plasma components were labeled using the ISBT128 coding system and were stored below minus 18/25 °C, in storage cabinets at the blood center. No nucleic acid amplification tests or pathogen inactivation processes were routinely performed.

### CP infusion

CP was delivered ready-for-use from the blood center to the pandemic intensive care units (ICUs) and each patient received two to three consecutive transfusions of 200 ml of A, B or O blood type (ABO)-compatible convalescent plasma (maximum of 600 ml of CP in total) over a period of 30 to 60 minutes, under supervision by the treating physician.

### Statistical analysis

The information collected was processed using the Statistical Package for Social Sciences (SPSS) for Windows, version 22.0 (IBM, Chicago, United States). The Shapiro-Wilk test was performed to check the normality assumption of the data. Parametric data were presented as mean ± standard deviation (SD), and intergroup significance was determined using Student’s t test. All the analyses were performed with the significance level set at P < 0.05.

### Ethics statement

The clinical research was approved by the local ethics committee (date: October 1, 2020; number: 2020/10/196). This study was conducted in conformity with the principles outlined in the Helsinki Declaration.

## RESULTS

A total of 30 patients with COVID-19-related ARDS were included in this study. The mean age of the patients was 62.16 ± 9.51 years and 73% of them were male. Hypertension (43.3%) was the most common comorbid disease. Sixty percent of the patients had previously received hydroxychloroquine. Two patients (6.7%) had severe ARDS. Four patients (13.3%) needed invasive mechanical ventilation.

Eleven patients (36%) were treated with CP in addition to antiviral therapy, and these were compared with nineteen patients (63%) who did not received CP. CP infusion was applied on a mean ± SD of 3.82 ± 3.68 days, and a median of three days (range: 1-14), after symptom onset. The mean age, gender, comorbidities and symptoms were similar between the two groups (P > 0.05). There was no significant difference between the groups regarding the severity of ARDS and need for respiratory support on admission (Table 1). Nasal oxygen was administered to eight patients (42%) in the control group and two patients (18%) in the CP group. On the 14^th^ day of treatment, the mean values ± SD of the PaO_2_/FiO_2_ ratio were 227.94 ± 124.98 in the CP group and 294.34 ± 144.59 in the control group. There was no statistically significant difference between the groups (P = 0.722). The length of time taken for the CP group to be discharged from the ICU was statistically significantly longer than that of the control group.

**Table 1.** Demographic and clinical characteristics of patients

Characteristics	Convalescent plasma group n = 11 (%)	Control group n = 19 (%)	Total n = 30 (%)	P
**Age, mean (± SD)**	59.81 (± 9.58)	63.52 (± 9.45)	62.16 (± 9.51)	0.312
**Male**	10 (90.9)	12 (63.2)	22 (73.3)	0.098
**Symptoms**				
Fever	8 (72.7)	10 (52.6)	18 (60.0)	0.279
Cough	5(45.5)	14 (73.7)	19 (63.3)	0.122
Dyspnea	8 (72.7)	13 (68.4)	21(70.0)	0.804
**Duration of symptoms, mean (± SD)**	3.82 (± 3.68)	4.68 (± 2.31)	4.37 (± 2.83)	0.430
**Comorbidities**				
Diabetes mellitus	2 (18.2)	8 (42.1)	10 (33.3)	0.180
Hypertension	3 (27.3)	10 (52.6)	13 (43.3)	0.177
Chronic obstructive pulmonary disease	2 (18.2)	3 (15.8)	5 (16.7)	0.865
**Antiviral treatment before convalescent plasma**				
Favipiravir	11 (100)	19 (100)	30 (100)	1.000
Hydroxychloroquine	9 (81.8)	9 (47.4)	18 (60.0)	0.143
Azithromycin	9 (81.8)	13 (68.4)	22 (73.3)	0.424
**Severity of ARDS**				
Mild	6 (54.5)	11 (57.9)	17 (56.7)	1.000
Moderate	4 (36.4)	7 (36.8)	11 (36.7)	1.000
Severe	1 (9.1)	1 (5.3)	2 (6.7)	0.552
PaO_2_/FiO_2_ on CP infusion day, mean (± SD)	205.34 (± 52.41)	212.04 (± 47.34)	209.59 (± 48.47)	0.214
**Respiratory support**				
Only nasal O_2_	2 (18.2)	8 (42.1)	10 (33.3)	0.180
High-flow O_2_	6 (54.5)	8 (42.1)	14 (46.7)	0.510
Invasive mechanical ventilation	1 (9.1)	3 (15.8)	4 (13.3)	0.603
**Prognosis**				
Discharge from intensive care unit up to 14^th^ day after CP	4 (36.4)	15 (78.9)	19 (63.3)	0.027
PaO_2_/FiO_2_ on 14^th^ day after CP, median (min-max)	227.94 (± 124.98)	294.34 (± 144.59)	269.99 (± 139.36)	0.722
Mortality up to 14^th^ day	1 (9.1)	3 (15.8)	4 (13.3)	0.603

SD = standard deviation; ARDS = acute respiratory distress syndrome; CP = convalescent plasma; min-max = minimum-maximum.

The laboratory findings were not significantly different between the groups on the day of CP infusion (P > 0.05) (Table 2).

**Table 2.** Laboratory test results on the day of convalescent plasma infusion

Laboratory tests, mean (± SD)	Control group, n = 19	Convalescent plasma group, n = 11	Total, n = 30	P
White blood cell count × 10^9^/l	7.52 (± 3.69)	8.21 (± 1.83)	7.77 (± 3.11)	0.504
Lymphocyte count × 10^9^/l	0.76 (± 0.45)	0.82 (± 0.15)	0.78 (± 0.37)	0.620
Creatinine, mg/dl	0.83 (± 0.35)	1.50 (± 1.65)	1.07 (± 1.06)	0.213
Aspartate aminotransferase, U/l	32.26 (± 13.01)	38.81 (± 24.68)	34.66 (± 18.04)	0.346
Alanine aminotransferase, U/l	26.42 (± 11.95)	26.54 (± 7.00)	26.46 (± 10.27)	0.972
Lactate dehydrogenase, U/l	383.47 (± 118.36)	435.64 (± 159.80)	402.60 (± 134.74)	0.315
Procalcitonin, µg/ml	3.50 (± 10.00)	2.56 (± 1.18)	3.16 (± 7.92)	0.760
C-reactive protein, mg/dl	154.44 (± 105.81)	141.63 (± 80.92)	149.75 (± 96.16)	0.732
Ferritin, µg/ml	1377.36 (± 1639.60)	1140.72 (± 315.77)	1290.60 (± 1310.12)	0.642
D- dimer, µg/ml	2451.89 (± 2789.59)	2000.72 (± 562.63)	2286.46 (± 2240.40)	0.505
Fibrinogen, mg/l	6278.42 (± 1771.96)	6316.00 (± 454.12)	6292.20 (± 1421.38)	0.931

SD =standard deviation.

On the 14^th^ day of treatment, the laboratory findings regarding leukocyte and lymphocyte counts and the levels of acute-phase reactants were similar between the two groups (P > 0.05) (Table 3). One intubated patient died on the 7^th^ day after CP infusion. Three patients in the control group were intubated, of whom one became extubated on the 4^th^ day.

**Table 3.** Laboratory test results on the 14^th^ day after convalescent plasma infusion

Laboratory tests, mean (± SD)	Control group, n = 19	Convalescent plasma group, n = 11	Total	P
White blood cell count ×10^9^/l	7.75 (± 4.74)	8.30 (± 2.91)	7.95 (± 4.12)	0.735
Lymphocyte count ×10^9^/l	0.92 (± 0.46)	0.87 (± 0.46)	0.90 (± 0.45)	0.784
Creatinine, mg/dl	1.55 (± 2.24)	1.40 (± 0.33)	1.50 (± 1.77)	0.819
Aspartate aminotransferase, U/l	32.94 (± 26.83)	26.90 (± 7.64)	30.73 (± 21.81)	0.475
Alanine aminotransferase, U/l	27.26 (± 19.29)	38.63 (± 21.98)	31.43 (± 20.70)	0.150
Lactate dehydrogenase, U/l	381.63 (± 134.83)	298.91 (± 127.38)	351.30 (± 136.10)	0.110
Procalcitonin, µg/ml	1.29 (± 1.80)	0.43 (± 0.83)	0.97 (± 1.56)	0.149
C-reactive protein, mg/dl	77.52 (± 75.76)	42.38 (± 31.01)	64.64 (± 64.73)	0.087
Ferritin, µg/ml	1760.15 (± 3716.18)	989.90 (± 1074.77)	1477.73 (± 3018.70)	0.510
D- dimer, µg/ml	3328.68 (± 4415.80)	2365.00 (± 1938.66)	2975.33(± 3690.81)	0.500
Fibrinogen, mg/l	5879.47 (± 1042.11)	5105.90 (± 1397.66)	5595.83 (± 1221.24)	0.095

SD = standard deviation.

None of the patients developed any side effects either during or after CP infusion.

## DISCUSSION

In this study, we retrospectively evaluated the effect of CP on the survival of patients with ARDS due to COVID-19 pneumonia. We observed that CP treatment did not improve the clinical or laboratory findings. Also, it had no effect regarding improvement of survival, i.e. regarding lowering mortality. Also, the time taken to be discharged from intensive care was longer. On the 14^th^ day, the PaO_2_/FiO_2_ ratio was lower in the CP group. Discharge from the intensive care unit may have been delayed due to the longer time taken for recovery of the oxygenation level.

The evidence of effectiveness of CP for treating COVID-19 is limited. There are quite a few different results regarding the effectiveness of CP. Firstly, Ye et al. observed that CP was effective in decreasing the viral load in non-severe cases. They also reported that patients with pneumonia had radiological improvements, but none of their cases were COVID-19-related ARDS.^8^ In a retrospective propensity-score matched-control study, Liu et al. assessed the efficacy of CP among severe COVID-19 patients. A significant reduction in oxygen requirements (adjusted odds ratio [OR] 0.86; P = 0.025) and an improvement in survival (adjusted hazard ratio [HR] 0.34; P = 0.027) were observed among patients treated with CP, in comparison with controls.^14^ In a one-arm multicenter interventional study, Perotti et al. observed the effects of CP infusion among 46 severe COVID-19 patients over a short period (seven days). Improvements in clinical and chest radiogram severity, laboratory test values (C-reactive protein, ferritin and lactate dehydrogenase) and functional respiratory parameters (PaO_2_/FiO_2_) were observed at the seven-day follow-up. A significant reduction in mortality rate (from 15% to 6.5%) was observed through comparing the mortality data of the present study with those of a historical cohort group.^15^

In contrast, in another study, the 28-day mortality rate among 52 critically ill patients with COVID-19 who were treated with CP in addition to standard therapy did not differ regarding clinical recovery, compared with the control group. However, patients with severe ARDS (PaO_2_/FiO_2_ ≤ 100; PEEP ≥ 5 cmH_2_O) were not included in that randomized study.^16^ An open-label randomized clinical trial on CP versus standard care, named the ConCOVID study, was conducted in the Netherlands. The trial was halted prematurely, when the baseline SARS-CoV-2 neutralizing antibody titers of the participants and the CP units transfused were found to be comparable, thus challenging the potential benefit of CP among the patients included in that study. In any case, no differences in mortality (P = 0.95), time spent in hospital (P = 0.68) or disease severity at day 15 (P = 0.58) were observed between the study arms.^17^

However, it should be noted that those studies enrolled patients with severe COVID-19 but did not focus on the effectiveness of CP for treating ARDS.

There were some limitations to our study. Firstly, its retrospective design and small number of patients was an important limitation. In addition, the inability to measure neutralizing antibodies in this study, in which we evaluated patients who we followed up in the early period of the pandemic, was another limitation.

## CONCLUSION

In our study, in which we investigated the effectiveness of CP for treating patients with ARDS due to COVID-19, we found that CP was not effective with regard to decreasing mortality.

Further prospective controlled studies are needed in order to evaluate the effectiveness of CP for treating patients with COVID-19-related ARDS.
